# Hypertension, Cardiovascular Risk Factors, and Uterine Fibroid Diagnosis in Midlife

**DOI:** 10.1001/jamanetworkopen.2024.6832

**Published:** 2024-04-16

**Authors:** Susanna D. Mitro, Lauren A. Wise, L. Elaine Waetjen, Catherine Lee, Eve Zaritsky, Siobán D. Harlow, Daniel H. Solomon, Rebecca C. Thurston, Samar R. El Khoudary, Nanette Santoro, Monique M. Hedderson

**Affiliations:** 1 Division of Research, Kaiser Permanente Northern California, Oakland; 2 Department of Epidemiology, Boston University School of Public Health, Boston, Massachusetts; 3 Department of Obstetrics and Gynecology, University of California Davis School of Medicine, Davis; 4 Department of Obstetrics and Gynecology, Kaiser Permanente Northern California, Oakland; 5 Department of Epidemiology, School of Public Health, University of Michigan, Ann Arbor; 6 Brigham and Women’s Hospital, Boston, Massachusetts; 7 Department of Psychology and Psychiatry, University of Pittsburgh Graduate School of Public Health, Pittsburgh, Pennsylvania; 8 Department of Epidemiology, University of Pittsburgh Graduate School of Public Health, Pittsburgh, Pennsylvania; 9 Department of Obstetrics and Gynecology, University of Colorado School of Medicine, Aurora

## Abstract

**Importance:**

Fibroids are benign neoplasms associated with severe gynecologic morbidity. There are no strategies to prevent fibroid development.

**Objective:**

To examine associations of hypertension, antihypertensive treatment, anthropometry, and blood biomarkers with incidence of reported fibroid diagnosis in midlife.

**Design, Setting, and Participants:**

The Study of Women’s Health Across the Nation is a prospective, multisite cohort study in the US. Participants were followed-up from enrollment (1996-1997) through 13 semiannual visits (1998-2013). Participants had a menstrual period in the last 3 months, were not pregnant or lactating, were aged 42 to 52 years, were not using hormones, and had a uterus and at least 1 ovary. Participants with prior fibroid diagnoses were excluded. Data analysis was performed from November 2022 to February 2024.

**Exposures:**

Blood pressure, anthropometry, biomarkers (cholesterol, triglycerides, and C-reactive protein), and self-reported antihypertensive treatment at baseline and follow-up visits were measured. Hypertension status (new-onset, preexisting, or never [reference]) and hypertension treatment (untreated, treated, or no hypertension [reference]) were categorized.

**Main Outcomes and Measures:**

Participants reported fibroid diagnosis at each visit. Discrete-time survival models estimated hazard ratios (HRs) and 95% CIs for associations of time-varying hypertension status, antihypertensive treatment, anthropometry, and biomarkers with incident reported fibroid diagnoses.

**Results:**

Among 2570 participants without a history of diagnosed fibroids (median [IQR] age at screening, 45 [43-48] years; 1079 [42.1%] college educated), 526 (20%) reported a new fibroid diagnosis during follow-up. Risk varied by category of hypertension treatment: compared with those with no hypertension, participants with untreated hypertension had a 19% greater risk of newly diagnosed fibroids (HR, 1.19; 95% CI, 0.91-1.57), whereas those with treated hypertension had a 20% lower risk (HR, 0.80; 95% CI, 0.56-1.15). Among eligible participants with hypertension, those taking antihypertensive treatment had a 37% lower risk of newly diagnosed fibroids (HR, 0.63; 95% CI, 0.38-1.05). Risk also varied by hypertension status: compared with never-hypertensive participants, participants with new-onset hypertension had 45% greater risk of newly diagnosed fibroids (HR, 1.45; 95% CI, 0.96-2.20). Anthropometric factors and blood biomarkers were not associated with fibroid risk.

**Conclusions and Relevance:**

Participants with untreated and new-onset hypertension had increased risk of newly diagnosed fibroids, whereas those taking antihypertensive treatment had lower risk, suggesting that blood pressure control may provide new strategies for fibroid prevention.

## Key Points

**Question:**

Are study-measured blood pressure, antihypertensive treatment, and cardiovascular risk factors (anthropometry and biomarkers) associated with incidence of fibroids?

**Findings:**

In this cohort study of 2570 individuals at midlife, participants with untreated and new-onset hypertension had increased risk of newly self-reported fibroids, whereas those taking antihypertensive treatment had lower risk. Anthropometric factors and blood biomarkers were not associated with risk of newly reported fibroids.

**Meaning:**

These findings motivate investigation into the mechanisms underlying fibroids and may lead to new strategies for their prevention.

## Teaser

This cohort study examines associations of hypertension, antihypertensive treatment, anthropometry, and blood biomarkers with incidence of reported uterine fibroid diagnosis in midlife.

## Introduction

Uterine fibroids are benign, hormonally responsive tumors that occur in 70% to 80% of people with uteruses by age 50 years, approximately one-half of whom have clinically relevant disease.^1^ A high-risk time for fibroid diagnosis begins around age 40 years.^2^ Although fibroids are common and cause debilitating symptoms, including pain and bleeding,^3^ no strategies are available to prevent them.

Growing evidence suggests that hypertension and other cardiovascular risk factors (including cholesterol levels, anthropometric measurements, carotid intima-media thickness, ankle-brachial index, and body composition) may be associated with fibroids.^4^ However, most studies have been cross-sectional,^5,6,7,8,9,10,11,12,13^ precluding analysis of temporality. Evidence from prospective studies is limited. One study^14^ reported a positive association between diastolic blood pressure and risk of fibroid diagnosis, whereas another^15^ reported that hypertension, but not heart attack or stroke, was associated with greater risk of fibroid surgery. A third study^16^ linked untreated hypertension with ultrasonography-confirmed fibroids only and treated hypertension with risk of hysterectomy-confirmed fibroids only. These prior prospective studies used self-reported blood pressure, hypertension diagnosis, and antihypertensive use and, thus, may be subject to reporting errors. If blood pressure affects fibroid risk, hypertension control may have the added benefit of preventing fibroid development or growth.

Prospective longitudinal studies with criterion-standard risk factor ascertainment are needed to elucidate potential associations between hypertension, markers of cardiovascular risk, and fibroids. Using data from a racially and ethnically diverse prospective cohort, Study of Women’s Health Across the Nation (SWAN), we examined whether measured hypertension, antihypertensive treatment, and other cardiovascular risk factors were associated with new fibroid diagnosis in midlife, when fibroids often become clinically apparent.

## Methods

SWAN is a multisite cohort study that enrolled 3302 participants between 1996 and 1997.^17^ Eligible participants had a menstrual period in the 3 months before enrollment, were not pregnant or lactating, were aged 42 to 52 years, were not using hormones, and had a uterus and at least 1 ovary. Recruitment details have been published elsewhere.^18^ Protocols were approved by institutional review boards at each participating institution. Participants provided written informed consent at each study visit. This manuscript follows Strengthening the Reporting of Observational Studies in Epidemiology (STROBE) reporting guideline for observational studies.

### Fibroid Diagnosis

Fibroid diagnosis was reported at baseline (visit 0, 1996-1997) and longitudinally through visit 13 (2011-2013, at which point nearly all participants were postmenopausal). Follow-up visits occurred approximately once per year from 1998 to 2008 (visits 1-11); visit 12 was in 2010 to 2011. At baseline participants were asked, “Has a doctor, nurse practitioner or other health care provider ever told you that you had fibroids, benign growths of the uterus or womb?” In visits 1 to 13, participants were asked, “Since your last study visit, have you had fibroids (benign growths in the uterus or womb)?” Self-reported fibroid diagnosis has been shown to have high specificity.^19^ We excluded participants who reported a history of fibroids at enrollment (670 participants [20.3%]) or were missing baseline fibroid status (62 participants [1.9%]). eTable 1 in Supplement 1 compares participants with and without fibroids at baseline. eFigure 1 in Supplement 1 shows a timeline and sample sizes for each visit.

### Measured Blood Pressure, Hypertension, and Anthropometry

At each visit, study staff measured blood pressure with a standardized protocol: 2 readings from the right arm after the participant had been seated for 5 minutes. We averaged the measured values. We defined hypertension as study-measured systolic blood pressure 130 mm Hg or higher or diastolic blood pressure 80 mm Hg or higher.^20^ Study staff also measured participant waist and hip circumference, height, and weight, and calculated waist-to-hip ratio and body mass index (BMI; calculated as weight in kilograms divided by height in meters squared).

### Blood-Based Biomarkers of Cardiovascular Disease Risk

We quantified high-sensitivity C-reactive protein (hsCRP), total cholesterol, high-density lipoprotein cholesterol, low-density lipoprotein cholesterol, and triglycerides in fasting blood samples (plasma or serum). hsCRP was quantified at baseline and visits 1, visits 3 to 7, and visits 9 to 12. Lipids were quantified at baseline and visits 1, 2 to 7, 9, 12, and 13. The study laboratory changed between visits 7 and 9, so concentrations were calibrated to ensure consistency. Additional details of the assays and calibration procedures are in the eAppendix in Supplement 1.

### Hypertension Treatment

We created a 3-category hypertension treatment variable to evaluate associations of fibroid diagnosis risk with treated and untreated hypertension. The hypertension treatment variable was updated at each visit and compared (1) participants with no antihypertensive treatment and measured normotension (no hypertension [reference group]) with (2) participants with no antihypertensive treatment and measured hypertension (untreated hypertension) and (3) participants receiving antihypertensive treatment regardless of measured blood pressure (treated hypertension).

### Hypertension Status

We also created a 3-category, time-varying hypertension status variable to capture associations of fibroid diagnosis risk with hypertension onset. The hypertension status variable used information from current and prior visits and compared (1) participants who had never reported antihypertensive use and never had measured hypertension (ie, systolic blood pressure ≥130 mm Hg and diastolic blood pressure ≥80 mm Hg) (never hypertensive [reference group]) with (2) participants who had reported antihypertensive treatment or had measured hypertension at past visits (preexisting hypertension) and (3) participants who reported antihypertensive treatment or had measured hypertension for the first time at the current visit (new-onset hypertension).

### Antihypertensive Medication Use

Participants reported all medication used at each visit, and study staff categorized each medication by mechanism. Antihypertensive medications included α-blockers, β-blockers, calcium channel blockers, angiotensin-converting enzyme (ACE) inhibitors, angiotensin II receptor blockers, and diuretics. Participants who reported use of antihypertensive medication in the current visit or in a prior visit were considered to be using antihypertensive medication; however, the type of antihypertensive medication used was updated at each visit and reflected current use only.

Models of time-varying antihypertensive treatment vs no treatment were conducted among a subpopulation of participants eligible to be treated with antihypertensives (624 participants at baseline; using antihypertensive medication; having systolic blood pressure ≥140 or diastolic blood pressure ≥90 mm Hg; or having systolic blood pressure ≥130 or diastolic blood pressure ≥80 mm Hg and at least 1 risk factor [age ≥65 years, history of cardiovascular event, 10-year atherosclerotic cardiovascular disease risk score ≥10%,^21^ or diabetes]) (eFigure 1 in Supplement 1).^20^ We also evaluated time-varying treatment with ACE inhibitors specifically, because of evidence suggesting that ACE inhibitors may be associated with lower fibroid incidence^22^ (ACE inhibitors were 28%-34% of antihypertensive medications used across visits). eTable 2 in Supplement 1 describes the subpopulation eligible for antihypertensive treatment.

### Covariates

Participants self-reported their age, educational attainment, race and ethnicity, smoking status, and parity. To identify race and ethnicity, participants were asked their primary group and told to choose 1 group if identifying as multiple races and ethnicities. Data on race and ethnicity are included in this analysis because Black women experience elevated risk of both fibroids and hypertension compared with White women.^23,24^

Because fibroids are diagnosed by radiological tests or physical examinations, they are more likely to be identified in participants with greater health care engagement. We operationalized health care utilization with variables that may reflect healthy behaviors, health care seeking, and health care access,^25^ such as multivitamin use, calcium supplementation, annual times spoken to a health care practitioner, and recency of last mammogram. To explore the role of gynecologic symptoms and care on likelihood of fibroid detection, we used recency of last pelvic examination, abnormal bleeding, and pelvic pain. We categorized menopausal status using data on menstrual bleeding patterns, pregnancy and/or breastfeeding, hormone use, and hysterectomy and/or oophorectomy.^26^ All variables except baseline age, education, site, and race and ethnicity were updated at each visit.

### Statistical Analysis

Data analysis was performed from November 2022 to February 2024. We used discrete-time survival models (with a complementary log-log link) to estimate hazard ratios (HRs) and 95% CIs for the associations of hypertension, anthropometry, blood biomarkers, and new reported fibroid diagnosis.^27^ We censored participants at menopause (because it is unusual for fibroids to develop after menopause),^28^ hysterectomy, loss to follow-up, or visit 13 (last visit with fibroid data), whichever occurred first. One site paused data collection for several years after visit 5, so we censored participants from that site at the pause. When participants had missing data due to a missed visit or questionnaire changes, we imputed the last observed value. Anthropometry, blood-based biomarkers, and hypertension variables were modeled separately to avoid collinearity.

Confounder selection was based on published literature indicating associations between each covariate and both fibroids and blood pressure and a directed acyclic graph (eFigure 2 in Supplement 1). We ran unadjusted models and models adjusted for the baseline value of the exposure, age,^1^ study site, educational attainment (less than high school, high school, some college and/or technical school, college graduate, or postgraduate),^28,29^ self-reported race and ethnicity (Black, Chinese, Hispanic, Japanese, or White),^29,30^ smoking status (current smoker vs nonsmoker),^31,32^ menopause status (perimenopausal, premenopausal or pregnant or breastfeeding, or unknown due to hormone use),^30,33^ parity (0, 1-2, ≥3 births),^30^ and BMI (continuous)^34,35^ (minimally adjusted models). Finally, we ran models including all covariates in the minimally adjusted model plus time-varying health care utilization variables reflecting behavior since the prior visit: times spoken to a health care practitioner (0, 1-2, or ≥3 times), use of multivitamins (yes or no), use of calcium supplements (yes or no), and mammogram (yes or no) (health care–adjusted models).

We further adjusted blood pressure models for antihypertensive medication use, and cholesterol and triglyceride models for statin use. Models of antihypertensive treatment vs no treatment were restricted to participants eligible to receive antihypertensive treatment (eTable 2 in Supplement 1).

Consistent with recommendations issued by the American Statistical Association,^36,37,38^ we interpret results according to the magnitude and direction of point estimates, width of 95% CIs, and pattern of findings across models, rather than statistical significance testing. Analyses were conducted using SAS statistical software 9.4 (SAS Institute).

In sensitivity analyses, to explore the role of gynecological symptoms or care, we repeated analyses restricted to participants who reported a pelvic examination, abnormal bleeding, or pelvic pain since the prior visit. To assess the effect of carrying forward observations to fill missing values, we repeated the main analysis without filling missing values (complete case analysis). Finally, we repeated analyses without censoring at menopause, and without adjusting for baseline values of exposure variables.

## Results

The eligible population of 2570 participants had a median (IQR) age of 45 (43-48) years. With regard to race and ethnicity, 644 (25.1%) were Black, 212 (8.3%) were Chinese, 235 (9.1%) were Hispanic, 223 (8.7%) were Japanese, and 1256 (48.9%) were White. A total of 1079 participants (42.1%) had a college education or greater, and 1398 (54.8%) were premenopausal (Table 1). Compared with participants with a history of fibroids, eligible participants were less likely to be Black (644 participants [25.1%] vs 284 participants [42.4%]), to have BMI 30 or greater (630 participants [25.7%] vs 195 participants [29.8%]), and to use antihypertensive medication (333 participants [13.0%] vs 120 participants [17.9%]) (eTable 1 in Supplement 1). Over follow-up, 526 participants (20% of eligible) reported a new diagnosis of fibroids.

**Table 1.** Baseline Characteristics of Eligible Participants in the Study of Women’s Health Across the Nation Cohort, 1996-2013

Characteristics	Participants, No. (%) (N = 2570)
Age at screening, median (IQR), y	45 (43-48)
Race and ethnicity	
Black	644 (25.1)
Chinese	212 (8.3)
Hispanic	235 (9.1)
Japanese	223 (8.7)
White	1256 (48.9)
Education^a^	
Less than high school	205 (8.0)
High school	468 (18.3)
Some college or technical school	806 (31.5)
College graduate	510 (19.9)
Postgraduate	569 (22.2)
Smoking status^b^	
Never	1479 (58.0)
Past	629 (24.7)
Current	443 (17.4)
Body mass index^c^	
<25.0	1175 (48.1)
25.0-29.9	640 (26.2)
30.0-34.9	326 (13.3)
≥35	304 (12.4)
Parity^d^	
0	422 (16.5)
1-2	1292 (50.4)
≥3	850 (33.2)
Menopause status^e^	
Premenopausal	1398 (54.8)
Early perimenopausal	1154 (45.2)
Blood pressure medication use	333 (13.0)
Angiotensin-converting enzyme inhibitor use	106 (4.1)
Statin use	19 (0.7)
Diabetes	105 (4.1)
Measured hypertension^f^	1020 (39.8)
Told you had high blood pressure^g^	472 (18.4)
Atherosclerotic cardiovascular disease risk score, median (IQR), %^h^	0.9 (0.5-1.8)
Take multivitamins^i^	1284 (51.0)
Spoken to health care practitioner this year^j^	2129 (84.0)

^a^ Data were missing for 12 participants.

^b^ Data were missing for 19 participants.

^c^ Body mass index is calculated as weight in kilograms divided by height in meters squared. Data were missing for 125 participants.

^d^ Data were missing for 6 participants.

^e^ Data were missing for 18 participants.

^f^ Data were missing for 7 participants.

^g^ Data were missing for 5 participants.

^h^ Data were missing for 24 participants.

^i^ Data were missing for 54 participants.

^j^ Data were missing for 35 participants.

We found no association between continuous blood pressure and new reported fibroid diagnosis (Table 2). However, participants with new-onset hypertension had a 45% higher risk of new reported fibroid diagnosis (HR, 1.45; 95% CI, 0.96-2.20) compared with never-hypertensive participants, whereas those with preexisting hypertension did not have higher risk. Similarly, participants with untreated hypertension had a 19% (HR, 1.19; 95% CI, 0.91-1.57) greater risk of new reported fibroid diagnosis compared with those with no hypertension, whereas those with treated hypertension had a 20% lower risk (HR, 0.80; 95% CI, 0.56-1.15).

**Table 2.** Longitudinal Discrete Survival Models Estimating the Risk of Newly Diagnosed Fibroids Associated With Each Cardiovascular Risk Factor in the Study of Women’s Health Across the Nation Cohort

Factor	HR (95% CI)		
	Crude	Minimally adjusted^a^	Health care adjusted^a^
Blood pressure and medication			
Systolic blood pressure (per 10 mm Hg)	1.02 (0.96-1.08)	1.00 (0.92-1.08)	1.01 (0.93-1.10)
Diastolic blood pressure (per 10 mm Hg)	1.03 (0.93-1.13)	0.96 (0.85-1.09)	0.98 (0.87-1.12)
Antihypertensive medication use (among eligible subset)^b^			
No antihypertensive use	1 [Reference]	1 [Reference]	1 [Reference]
Any antihypertensive use	1.10 (0.72-1.68)	0.93 (0.57-1.53)	0.63 (0.38-1.05)
Angiotensin-converting enzyme inhibitor use	1.00 (0.59-1.70)	0.80 (0.42-1.52)	0.52 (0.27-1.00)
Hypertension status^c^			
Never hypertensive	1 [Reference]	1 [Reference]	1 [Reference]
Preexisting hypertension	1.22 (0.99-1.51)	0.99 (0.78-1.25)	0.93 (0.73-1.18)
New-onset hypertension	1.84 (1.23-2.76)	1.56 (1.03-2.35)	1.45 (0.96-2.20)
Hypertension treatment			
No hypertension	1 [Reference]	1 [Reference]	1 [Reference]
Untreated hypertension	1.23 (0.96-1.58)	1.13 (0.85-1.48)	1.19 (0.91-1.57)
Treated hypertension	1.29 (1.01-1.63)	1.02 (0.71-1.47)	0.80 (0.56-1.15)
Anthropometry			
Body mass index (per 5 points)^d^	1.05 (0.98-1.12)	0.95 (0.80-1.13)	0.91 (0.77-1.08)
<25.0	1 [Reference]	1 [Reference]	1 [Reference]
25.0-29.9	1.02 (0.79-1.31)	0.82 (0.62-1.09)	0.78 (0.59-1.04)
30.0-34.9	1.06 (0.79-1.42)	0.76 (0.52-1.10)	0.70 (0.48-1.02)
≥35	1.14 (0.87-1.50)	0.67 (0.39-1.13)	0.56 (0.33-0.96)
Waist circumference (per centimeter)	1.00 (1.00-1.01)	1.01 (0.99-1.03)	1.01 (0.99-1.03)
Waist-to-hip ratio (per 0.1 unit)	1.10 (0.96-1.25)	1.35 (1.06-1.71)	1.29 (1.02-1.63)
Biomarkers			
Total cholesterol (per 10 mg/dL)	0.99 (0.96-1.02)	0.98 (0.94-1.02)	0.98 (0.94-1.02)
Low-density lipoprotein cholesterol (per 10 mg/dL)	1.00 (0.97-1.03)	0.99 (0.94-1.04)	0.99 (0.94-1.04)
High-density lipoprotein cholesterol (per 10 mg/dL)	1.01 (0.95-1.08)	1.03 (0.92-1.17)	1.03 (0.92-1.16)
Triglycerides (per 10 mg/dL)	0.99 (0.98-1.01)	0.99 (0.97-1.01)	0.99 (0.97-1.01)
C-reactive protein (per doubling)	1.04 (0.99-1.09)	1.02 (0.95-1.10)	1.01 (0.94-1.09)

Abbreviation: HR, hazard ratio.

SI conversion factors: To convert high-density lipoprotein cholesterol to millimoles per liter, multiply by 0.0259; low-density lipoprotein cholesterol to millimoles per liter, multiply by 0.0259; triglycerides to millimoles per liter, multiply by 0.0113.

^a^ Minimally adjusted model was adjusted for baseline value of the exposure, age, education, race and ethnicity, site, time-varying smoking status, menopausal status, parity, and body mass index. The health care–adjusted model was also adjusted for time-varying times spoken to a health care practitioner, multivitamin receipt, calcium supplement receipt, and mammogram since prior visit. Blood pressure models additionally adjusted for blood pressure medication; cholesterol and triglyceride models additionally adjusted for statins.

^b^ Refers to participants eligible to use blood pressure medication (624 participants at baseline).

^c^ The never hypertensive group had no history of antihypertensive use or measured hypertension, the preexisting hypertension group previously reported antihypertensive use or had measured hypertension, and the new-onset hypertension group reported antihypertensive use or had measured hypertension for the first time at the current visit.

^d^ Body mass index is calculated as weight in kilograms divided by height in meters squared.

Participants eligible to use antihypertensive medication were more likely than the overall study population to be non-Hispanic Black and have BMI 30 or higher (eTable 2 in Supplement 1). Among participants eligible to use antihypertensive treatment, those using antihypertensive treatment had a 37% lower risk of new reported fibroid diagnosis (HR, 0.63; 95% CI, 0.38-1.05); reduction in risk was especially pronounced among participants using ACE inhibitors compared with those using no antihypertensive treatment (48% lower risk; HR, 0.52; 95% CI, 0.27-1.00) (Table 2).

Anthropometry, lipids, and hsCRP were not associated with risk of new reported fibroid diagnosis. For each 0.1-unit increase in waist-to-hip ratio, the risk of newly developed fibroids increased by 29% (HR, 1.29; 95% CI, 1.02-1.63), but there were no associations with BMI, waist circumference, lipids, or hsCRP (Table 2). In sensitivity analyses, the findings slightly attenuated in models restricted to participants who reported a pelvic examination, abnormal bleeding, or pelvic pain since the prior visit (63%-72% of participants); in models without carried forward observations to fill missing values; in models that did not censor at menopause (postmenopausal participants increased from 2% at visit 1 to 99% at visit 13); and in models without adjustment for baseline exposure values (eTables 3-6 in Supplement 1).

## Discussion

In this longitudinal cohort study with repeated measures of both cardiovascular risk factors and self-reported fibroid diagnosis, patients with new-onset hypertension had a 45% increased risk of newly reported fibroids, and those with untreated hypertension had a 19% increased risk. Participants who used antihypertensive medication had lower risks of newly reported fibroids: 20% lower risk compared with participants without hypertension, and 37% lower risk (for ACE inhibitors, 48% lower risk) compared with untreated participants eligible to use antihypertensive medication. These findings suggest that new hypertension is associated with elevated risk of first fibroid diagnosis at midlife, a high-risk time for both fibroid diagnosis and the development of cardiovascular risk.

Our finding that risk of new reported fibroid diagnosis was higher among participants with untreated vs treated hypertension, whereas continuous blood pressure was not associated with risk, adds important new insights to a conflicting literature. Both the Black Women’s Health Study, a prospective cohort of premenopausal Black women, and the Nurses’ Health Study II, a prospective cohort of nurses aged 25 to 42 years at baseline, found that higher blood pressure was associated with greater fibroid incidence regardless of antihypertensive use.^14,16^ We also found that new-onset hypertension carried greater risk of newly reported fibroids than ongoing hypertension, which differs from prior research suggesting either sustained elevation^14^ or no change^16^ in fibroid risk after hypertension diagnosis. Our findings may differ from prior literature for many reasons. Importantly, we relied on technician-measured blood pressure to ascertain hypertension status, thereby reducing misclassification; participants with untreated hypertension may not be aware of their hypertension. Also, previous studies less precisely ascertained hypertension onset, which may have attenuated the associations. Finally, prior cohort studies included a wider age range (eg, approximately 20-80 years in some cases^14,15,16^), possibly diluting associations. Nonetheless, our results add to the prospective evidence linking untreated hypertension to risk of newly clinically apparent fibroids.

Our related finding among participants eligible to use antihypertensive medication that those using antihypertensive medication had a 37% lower risk of newly reported fibroids, and those using ACE inhibitors in particular had 48% lower risk, is consistent with a recent commercial insurance database study that reported that ACE inhibitor use was associated with 32% reduced odds of new fibroid diagnosis.^22^ Preliminary evidence suggests that the renin-angiotensin-aldosterone pathway may explain a protective effect of ACE inhibitors: angiotensin II and aldosterone have been shown to cause fibroid cell proliferation in a rat cell line,^39,40^ and mixed evidence from human genetic studies^41^ suggests that fibroid risk is associated with angiotensin II receptor type 1 genetic sequence variants^42^ and angiotensin-converting enzyme insertion-deletion genetic sequence variants.^43,44^ The renin-angiotensin-aldosterone pathway deserves further exploration in light of our findings suggesting reduction of fibroid risk associated with ACE inhibitors.

Alternatively, associations between new-onset hypertension and newly reported fibroid diagnosis may be due to bias. For example, unmeasured comorbidities could increase both blood pressure and health care utilization, leading to incidental fibroid diagnosis; however, results were robust to adjustment for health care utilization and sensitivity analyses restricting to participants with a pelvic examination or symptoms. Reverse causation is also possible: a growing fibroid might increase blood pressure through vascular demands^4^ or altered systemic hemodynamics.^45^

Although a growing literature links cardiovascular risk factors to prevalent fibroids, nearly all studies have been cross-sectional in design. In this prospective study, we reported null associations between lipids, CRP, anthropometry (except waist-to-hip ratio), and incidence of fibroids. Prior studies have reported mixed findings for the association between cholesterol and fibroids, with some linking fibroids to higher low-density lipoprotein and lower high-density lipoprotein cholesterol,^7,11,13,46^ some suggesting the opposite,^47^ and others reporting null associations.^5,48,49^ The only prior study to investigate associations between CRP and fibroid prevalence reported null associations.^50^ Prior literature has reported nonlinear associations between BMI and fibroids (highest risk for BMI approximately 28-32) and both positive and null associations between waist-to-hip ratio and fibroids.^7,34,51,52,53^ Previous studies varied widely in design, which may also explain heterogeneity; for example, some relied on retrospective analyses (eg, lifetime fibroid occurrence),^5^ clinical convenience samples,^11^ or surgically confirmed cases.^54^ Our null findings in a longitudinal cohort study with criterion-standard biomarker measurements do not support an effect of lipids or CRP on new fibroid diagnosis in midlife participants.

Our analysis benefited from high-quality, repeated measurements of blood pressure, biomarkers, and anthropometry, enabling us to assess exposures independent of health care access. SWAN’s midlife participants and criterion-standard assessment of menopause status allowed us to focus on a high-risk life stage.^55^ Finally, we adjusted for a wide range of potential confounding factors, including health care utilization (an especially strong confounder of associations between medication use and fibroid diagnosis).

### Limitations

Our work also has limitations. First, we used self-reported fibroid diagnosis; therefore, asymptomatic fibroids, which may constitute more than one-half of cases,^2^ may be missed. Complete fibroid ascertainment (ie, repeated ultrasonography of asymptomatic participants) is difficult, and all prior prospective studies of hypertension and fibroids also relied on self-reported diagnosis.^14,15,16^ Self-reported fibroid diagnosis has high specificity^19^; sensitivity is estimated to be approximately 50% for women aged 35 to 49 years.^19^ Because fibroid misclassification is unlikely to vary by study-measured hypertension status and specificity is high, despite lower sensitivity, effect estimates are expected to be unbiased.^56^ On the other hand, if participants with study-measured hypertension had lower rates of missed fibroid diagnosis than those with normotension, the magnitude of our associations would be overstated. Second, the eligibility criteria may limit generalizability. Fibroids often develop before midlife, especially for Black women^28^; in this study, Black participants were more likely to be excluded because of prior fibroids. Findings are most applicable to those with a first fibroid diagnosis in midlife. Fibroids may grow slowly^57^ and may have been present but undiagnosed before hypertension onset. However, prospective associations between new-onset hypertension and fibroid diagnosis suggest that high blood pressure plays a role in fibroid growth to the point of clinical recognition. Participants using ACE inhibitors may differ from those using other antihypertensive medications or no medications; further investigation is needed to determine the causal effect of ACE inhibitor use on fibroid risk. Furthermore, we could not rule out unmeasured confounding but were able to adjust for multiple demographic and health care utilization variables.

## Conclusions

This study of a midlife cohort found that patients with untreated and new-onset hypertension had increased risk of newly reported fibroid diagnosis, whereas those taking antihypertensive medication had a reduced risk. Investigation into mechanisms and health implications is warranted; if the associations are causal, antihypertensive medication use where indicated may present an opportunity to prevent clinically apparent fibroid development at this high-risk life stage.

## Notes

Supplement 1. **eAppendix.** Assay Details for Cardiovascular Biomarkers
eReferences
**eFigure 1.** Study Timeline and Schematic **eFigure 2.** Directed Acyclic Graph (DAG) Showing the Relationships Between Each Confounder, the Exposure (in This Example, Blood Pressure) and the Outcome (Self-Reported Fibroid Diagnosis) **eTable 3.** Longitudinal Discrete Survival Models Estimate the Risk of Newly Diagnosed Fibroids Associated With Each Cardiovascular Risk Factor in the Study of Women’s Health Across the Nation (SWAN) Cohort **eTable 4.** Longitudinal Discrete Survival Models Estimate the Risk of Newly Diagnosed Fibroids Associated With Each Cardiovascular Risk Factor in the Study of Women’s Health Across the Nation (SWAN) Cohort **eTable 5.** Longitudinal Discrete Survival Models Estimate the Risk of Newly Diagnosed Fibroids Associated With Each Cardiovascular Risk Factor in the Study of Women’s Health Across the Nation (SWAN) Cohort **eTable 6.** Longitudinal Discrete Survival Models Estimate the Risk of Newly Diagnosed Fibroids Associated With Each Cardiovascular Risk Factor in the Study of Women’s Health Across the Nation (SWAN) Cohort

Supplement 2.
Data Sharing Statement
