# Supplementary material for: Hypertension, Cardiovascular Risk Factors, and Uterine Fibroid Diagnosis in Midlife
Source: JAMA Netw Open. 2024 Apr 16;7(4):e246832. doi: 10.1001/jamanetworkopen.2024.6832 (PMC11022113; doi:10.1001/jamanetworkopen.2024.6832)
Supplement: Supplement 1. — eAppendix. Assay Details for Cardiovascular Biomarkers eReferences eFigure 1. Study Timeline and Schematic eFigure 2. Directed Acyclic Graph (DAG) Showing the Relationships Between Each Confounder, the Exposure (in This Example, Blood Pressure) and the Outcome (Self-Reported Fibroid Diagnosis) eTable 3. Longitudinal Discrete Survival Models Estimate the Risk of Newly Diagnosed Fibroids Associated With Each Cardiovascular Risk Factor in the Study of Women’s Health Across the Nation (SWAN) Cohort eTable 4. Longitudinal Discrete Survival Models Estimate the Risk of Newly Diagnosed Fibroids Associated With Each Cardiovascular Risk Factor in the Study of Women’s Health Across the Nation (SWAN) Cohort eTable 5. Longitudinal Discrete Survival Models Estimate the Risk of Newly Diagnosed Fibroids Associated With Each Cardiovascular Risk Factor in the Study of Women’s Health Across the Nation (SWAN) Cohort eTable 6. Longitudinal Discrete Survival Models Estimate the Risk of Newly Diagnosed Fibroids Associated With Each Cardiovascular Risk Factor in the Study of Women’s Health Across the Nation (SWAN) Cohort [file jamanetwopen-e246832-s001.pdf]

Supplemental Online Content

Mitro SD, Wise LA, Waetjen LE, et al. Hypertension, cardiovascular risk factors, and uterine fibroid diagnosis in midlife. *JAMA Netw Open*. 2024;7(4):e246832. doi:10.1001/jamanetworkopen.2024.6832

eAppendix. Assay Details for Cardiovascular Biomarkers
eReferences
eFigure 1. Study Timeline And Schematic
eFigure 2. Directed Acyclic Graph (DAG) Showing the Relationships Between Each Confounder, the Exposure (in This Example, Blood Pressure) and the Outcome (Self-Reported Fibroid Diagnosis)
eTable 3. Longitudinal Discrete Survival Models Estimate the Risk of Newly Diagnosed Fibroids Associated With Each Cardiovascular Risk Factor in the Study of Women's Health Across the Nation (SWAN) Cohort
eTable 4. Longitudinal Discrete Survival Models Estimate the Risk of Newly Diagnosed Fibroids Associated With Each Cardiovascular Risk Factor in the Study of Women's Health Across the Nation (SWAN) Cohort
eTable 5. Longitudinal Discrete Survival Models Estimate the risk of Newly Diagnosed Fibroids Associated With Each Cardiovascular Risk Factor in the Study of Women's Health Across the Nation (SWAN) Cohort
eTable 6. Longitudinal Discrete Survival Models Estimate the Risk of Newly Diagnosed Fibroids Associated With Each Cardiovascular Risk Factor in the Study of Women's Health Across the Nation (SWAN) Cohort

This supplemental material has been provided by the authors to give readers additional information about their work.

© 2024 Mitro SD et al. *JAMA Network Open.*

eAppendix. Assay Details for Cardiovascular Biomarkers

CRP. CRP assay 1: From baseline to follow-up visit 7 (with the exception of visit 2, when CRP was not assayed), CRP was measured using an ultrasensitive rate immunonephelometric method with a lower limit of detection (LLD, also referred to as sensitivity) of 0.042 mg/L (BN100; Dade-Behring, Marburg, Germany). The intra-assay CV at CRP concentrations of 0.5 and 22.0 mg/L were 10–12% and 5–7%, respectively.

CRP Assay 2: Samples from follow-up visits 9 and 10 were assayed using a high sensitivity immunoassay (Alfa Wassermann, West Caldwell, NJ). The LLD was 0.13 mg/L, and the intra-assay CV at CRP concentrations of 0.85 and 8.62 mg/L were 4.0% and 0.7%, respectively.

CRP assay 3: For follow-up visit 12, CRP was assayed using the ACE *Ultra*Wide Range assay, a latex-enhanced turbidimetric *in vitro* immunoassay (Alfa Wassermann, West Caldwell, NJ). The LLD was 0.05 mg/L, and the intra-assay CV at CRP concentrations of 0.46 mg/L and 9.81 mg/L were 5.74% and 1.16%, respectively.

CRP assay 4: For approximately 25% of all samples run at visits 9 and 10, CRP was below the LLD of assay 3 (outlined above). In these instances, an additional sample was retrieved from the Repository and CRP was measured using the Human High Sensitivity CRP ELISA (R&D Systems, DCRP00), a quantitative sandwich enzyme immunoassay. The LLD for this assay was 0.010 ng/ml (or 0.00001 mg/L). All values above the LLD for the 3 original assays were calibrated to the high sensitivity ELISA by simultaneously assaying 600 paired samples (representing the full range of results from each of the original 3 assays) and the high sensitivity assay; thus, there were 200 paired samples for each original vs. high sensitivity ELISA. After calibration, correlations between the first 3 CRP assays and the fourth, high sensitivity ELISA were $\geq$0.94.

Total cholesterol. From baseline to visit 7, cholesterol was measured using an automated cholesterol oxidase assay on a Hitachi 747-200 clinical analyzer using RAICHEM reagents and calibrators with target values assigned by the Centers for Disease Control Lipid Section. At visits 9, 12, and 13 the cholesterol esters were hydrolyzed by cholesterol esterase to cholesterol and free fatty acids. The cholesterol was converted to cholest-4-en-3-one by cholesterol oxidase in the presence of oxygen to form hydrogen peroxide. A colored complex was formed from hydrogen peroxide, 4-aminoantipyrine and phenol under the catalytic influence of peroxidase, and the absorbance of the complex was measured at 505/694 nm.

© 2024 Mitro SD et al. *JAMA Network Open.*

LDL cholesterol was calculated at al visits using the Friedewald equation (total cholesterol – (triglycerides / 5) – HDL cholesterol)[1] when triglycerides were below 400 mg/dL.

HDL cholesterol. From baseline to visit 7, HDL cholesterol was measured following precipitation of LDL and VLDL with heparin and manganese chloride by the modified Lipid Research Clinics procedure. HDL cholesterol in the supernate was measured by an automated cholesterol oxidase assay on a Hitachi 747-200 clinical analyzer using RAICHEM reagents, a low cholesterol level calibrator with target values assigned by the Centers for Disease Control Lipid Section, and including controls for low level cholesterol in addition to controls for HDL cholesterol. At visits 9, 12, and 13 HDL cholesterol was measured without prior separation, based on procedures developed by Izawa, Okada and Matsui.[2] Cholesterol from non-HDL particles was released and eliminated in the first step of the reaction, and cholesterol in HDL particles was released in the second step by detergent in R2. The HDL cholesterol was measured by a Trinder reaction.

Triglycerides. From baseline to visit 7, triglycerides were measured using an automated glycerol kinase enzymatic assay on a Hitachi 747-200 clinical analyzer using Technicon reagents and with controls with target values assigned by the Centers for Disease Control Lipid Section. Glycerol blanks were performed for any samples with triglycerides greater than 300 mg/dL. At visits 9, 12, and 13, triglycerides were measured using the ADVIA assay method: triglycerides were converted to glycerol and free fatty acids by lipase, and the glycerol was then converted to glycerol-3-phosphate by glycerol kinase followed by its conversion by glycerol-3-phosphate-oxidase to hydrogen peroxide. A colored complex was formed from hydrogen peroxide, 4-aminophenazone and 4-chlorophenol under the catalytic influence of peroxidase. The absorbance of the complex was measured as an endpoint reaction at 505/694 nm.

eReferences

1. Friedewald WT, Levy RI, Fredrickson DS. Estimation of the concentration of low-densiy lipoprotein cholesterol in plasma, without use of the preparative ultracentrifuge. *Clinical Chemistry.* 1972;18(6):499-502.
2. Izawa S, Okada M, Matsui H, Horita Y. A new direct method for measuring HDL-cholesterol which does not produce any biased values. *J Med Pharm Sci* 1997;37:1385-1388.

© 2024 Mitro SD et al. *JAMA Network Open.*

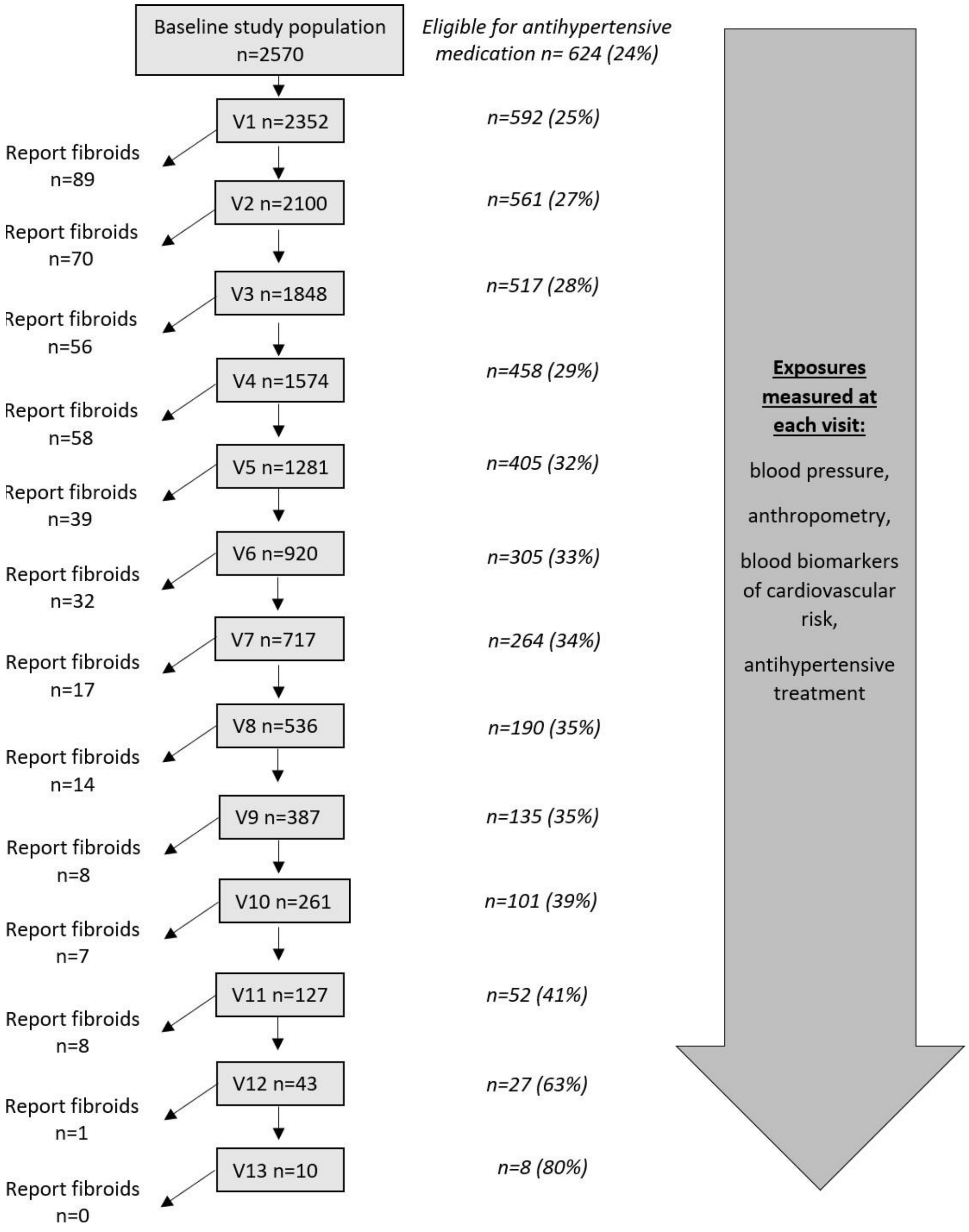

eFigure 1. Study Timeline And Schematic. The eligible population at each visit (i.e., pre- and perimenopausal without a prior fibroid diagnosis) is depicted in light grey squares. The number of eligible participants reporting a fibroid diagnosis at each visit is shown to the left. The number of participants eligible to receive antihypertensive medication is shown to the right.

© 2024 Mitro SD et al. *JAMA Network Open.*

eTable 1. Characteristics of Participants With (n=670) and Without (n=2570) Fibroids at Baseline

**Baseline characteristics**	**Ever had a fibroid at baseline (n=670)**	**Never had a fibroid at baseline (n=2570)**
Age at screener, median (IQR)	46 (44, 48)	45 (43, 48)
Race/ethnicity, n(%)		
Black	284 (42.4)	644 (25.1)
White	264 (39.4)	1256 (48.9)
Chinese	32 (4.8)	212 (8.3)
Hispanic	37 (5.5)	235 (9.1)
Japanese	53 (7.9)	223 (8.7)
Education, n(%)[1]		
<High school	29 (4.4)	205 (8.0)
High school	102 (15.4)	468 (18.3)
Some college/ technical school	231 (34.8)	806 (31.5)
College graduate	140 (21.1)	510 (19.9)
Postgraduate	161 (24.3)	569 (22.2)
Smoking status, n(%)[2]		
Never	380 (57.8)	1479 (58.0)
Past	165 (25.1)	629 (24.7)
Current	113 (17.2)	443 (17.4)
Body mass index, kg/m^2, n(%)[3]		
< 25.0	280 (42.7)	1175 (48.1)
25.0 – 29.9	181 (27.6)	640 (26.2)
30.0 – 34.9	112 (17.1)	326 (13.3)
≥ 35	83 (12.7)	304 (12.4)
Difficulty paying for basics, n(%)[4]		
Very hard	62 (9.3)	238 (9.3)
Somewhat hard	183 (27.4)	810 (31.6)
Not very hard	422 (63.3)	1515 (59.1)
Parity, n(%)[5]		
0	124 (18.5)	422 (16.5)
1-2	332 (49.6)	1292 (50.4)
3+	214 (31.9)	850 (33.2)
Years since last child was born, median (IQR)[6]	16 (11, 21)	15 (10, 19)
History of breastfeeding, n(%)	315 (57.7)	1399 (65.3)
Menopause status, n(%)[7]		
Premenopausal	318 (48.5)	1398 (54.8)
Early perimenopausal	338 (51.5)	1154 (45.2)
Ever used hormones (including birth control), n(%)[8]	112 (17.0)	260 (10.2)
Blood pressure medication use, n(%)	120 (17.9)	333 (13.0)
ACE-inhibitor use, n(%)	38 (5.7)	106 (4.1)
Statin use, n(%)	10 (1.5)	19 (0.7)
Diabetes, n(%)	47 (7.0)	105 (4.1)
Measured hypertension, n(%)[9]	269 (40.3)	1020 (39.8)
Told you had high blood pressure, n(%)[4]	168 (25.3)	472 (18.4)
ASCVD risk score (%), median (IQR)[2]	0.9 (0.5, 2.1)	0.9 (0.5, 1.8)
Take multivitamins, n(%)[10]	370 (56.4)	1284 (51.0)
Spoken to healthcare provider this year, n(%)[11]	604 (91.2)	2129 (84.0)

Missing:[1]19; [2]31; [3]139; [4]10; [5]6; [6]20;[7]32; [8]40; [9]9; [10]68; [11]43
Years since last child and history of breastfeeding calculated among parous participants only

© 2024 Mitro SD et al. *JAMA Network Open.*

eTable 2. Characteristics of Participants Eligible to Receive Antihypertensive Treatment (n=624)

**Baseline characteristics**	**Eligible to receive antihypertensive treatment (n=624)**	**Full study population (n=2570)**
Age at screener, median (IQR)	46 (44, 48)	45 (43, 48)
Race/ethnicity, n(%)		
Black	271 (43.4)	644 (25.1)
White	229 (36.7)	1256 (48.9)
Chinese	25 (4.0)	212 (8.3)
Hispanic	75 (12.0)	235 (9.1)
Japanese	24 (3.9)	223 (8.7)
Education, n(%)		
<High school	65 (10.5)	205 (8.0)
High school	133 (21.5)	468 (18.3)
Some college/ technical school	209 (33.7)	806 (31.5)
College graduate	100 (16.1)	510 (19.9)
Postgraduate	113 (18.2)	569 (22.2)
Smoking status, n(%)		
Never	341 (54.8)	1479 (58.0)
Past	145 (23.3)	629 (24.7)
Current	136 (21.9)	443 (17.4)
Body mass index, kg/m^2, n(%)		
< 25.0	155 (26.5)	1175 (48.1)
25.0 – 29.9	165 (28.2)	640 (26.2)
30.0 – 34.9	116 (19.8)	326 (13.3)
≥ 35	150 (25.6)	304 (12.4)
Difficulty paying for basics, n(%)		
Very hard	93 (14.9)	238 (9.3)
Somewhat hard	202 (32.4)	810 (31.6)
Not very hard	328 (52.7)	1515 (59.1)
Parity, n(%)		
0	91 (14.6)	422 (16.5)
1-2	292 (46.9)	1292 (50.4)
3+	240 (38.5)	850 (33.2)
Years since last child was born, median (IQR)	16 (12, 20)	15 (10, 19)
History of breastfeeding, n(%)	288 (54.1)	1399 (65.3)
Menopause status, n(%)		
Premenopausal	303 (48.9)	1398 (54.8)
Early perimenopausal	317 (51.1)	1154 (45.2)
Ever used hormones (including birth control), n(%)	72 (11.6)	260 (10.2)
Blood pressure medication use, n(%)	333 (53.4)	333 (13.0)
ACE-inhibitor use, n(%)	106 (17.0)	106 (4.1)
Statin use, n(%)	13 (2.1)	19 (0.7)
Diabetes, n(%)	75 (12.0)	105 (4.1)
Measured hypertension, n(%)	513 (82.2)	1020 (39.8)
Told you had high blood pressure, n(%)	345 (55.5)	472 (18.4)
ASCVD risk score (%), median (IQR)	2.3 (1.3, 4.6)	0.9 (0.5, 1.8)
Take multivitamins, n(%)	318 (52.0)	1284 (51.0)
Spoken to healthcare provider this year, n(%)	555 (90.4)	2129 (84.0)

Years since last child and history of breastfeeding calculated among parous participants only

© 2024 Mitro SD et al. *JAMA Network Open.*

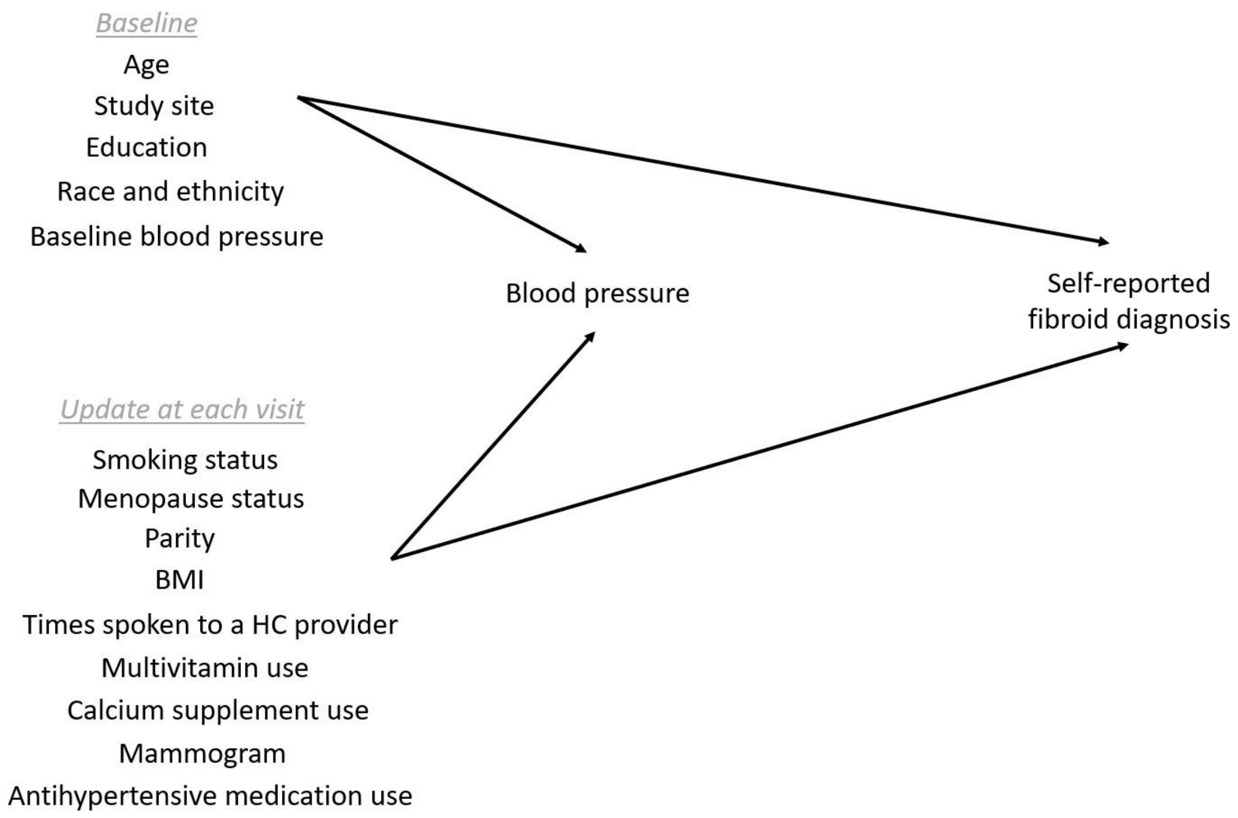

eFigure 2. Directed Acyclic Graph (DAG) Showing the Relationships Between Each Confounder, the Exposure (in This Example, Blood Pressure) and the Outcome (Self-Reported Fibroid Diagnosis). The exposure, outcome, and some confounders update at each study visit. At each study visit, discrete-time survival models tested the association between exposure and outcome, adjusting for visit-specific covariate levels, and pooled across visits.

© 2024 Mitro SD et al. *JAMA Network Open.*

eTable 3. Longitudinal Discrete Survival Models Estimate the Risk of Newly Diagnosed Fibroids Associated With Each Cardiovascular Risk Factor in the Study of Women's Health Across the Nation (SWAN) Cohort. The "Symptoms and screening" column represents regression models that are identical to the Healthcare- adjusted model but restricted to participants who reported a pelvic exam, abnormal bleeding, or pelvic pain since the prior visit (63-80% of eligible participants per visit (average 70%)).

	Healthcare-adjusted[1]	Symptoms and screening
**Characteristic**	HR (95%CI)	HR (95%CI)
**Blood pressure and medication**		
Systolic blood pressure (per 10-mm Hg)	1.01 (0.93, 1.10)	1.02 (0.94, 1.11)
Diastolic blood pressure (per 10-mm Hg)	0.98 (0.87, 1.12)	1.00 (0.88, 1.15)
Antihypertensive medication use (among eligible subset)[2]		
No antihypertensive use	*Reference*	*Reference*
Any antihypertensive use	0.63 (0.38, 1.05)	0.59 (0.35, 0.99)
ACE-inhibitor use	0.52 (0.27, 1.00)	0.54 (0.27, 1.04)
Hypertension status[3]		
Never hypertensive	*Reference*	*Reference*
Pre-existing hypertension	0.93 (0.73, 1.18)	0.96 (0.75, 1.23)
New-onset hypertension	1.45 (0.96, 2.20)	1.52 (1.00, 2.32)
Hypertension treatment		
No hypertension	*Reference*	*Reference*
Untreated hypertension	1.19 (0.91, 1.57)	1.26 (0.95, 1.67)
Treated hypertension	0.80 (0.56, 1.15)	0.85 (0.59, 1.23)
**Anthropometry**		
Body mass index (kg/m^2) (per 5-kg/m^2)	0.91 (0.77, 1.08)	0.95 (0.80, 1.13)
< 25.0	*Reference*	*Reference*
25.0 – 29.9	0.78 (0.59, 1.04)	0.82 (0.61, 1.11)
30.0 – 34.9	0.70 (0.48, 1.02)	0.77 (0.52, 1.14)
≥ 35	0.56 (0.33, 0.96)	0.64 (0.37, 1.13)
Waist circumference (per centimeter)	1.01 (0.99, 1.03)	1.02 (1.00, 1.04)
Waist-to-hip ratio (per 0.1-unit)	1.29 (1.02, 1.63)	1.41 (1.11, 1.80)
**Biomarkers**		
Total cholesterol (per 10-mg/dL)	0.98 (0.94, 1.02)	0.98 (0.94, 1.02)
LDL cholesterol (per 10-mg/dL)	0.99 (0.94, 1.04)	1.00 (0.95, 1.05)
HDL cholesterol (per 10-mg/dL)	1.03 (0.92, 1.16)	1.04 (0.92, 1.17)
Triglycerides (per 10-mg/dL)	0.99 (0.97, 1.01)	0.99 (0.97, 1.01)
C-reactive protein (per doubling)	1.01 (0.94, 1.09)	1.03 (0.95, 1.11)

[1]*Minimally-adjusted model adjusted for baseline value of the exposure, age, education, race/ethnicity, site; time-varying smoking status, menopausal status, parity, body mass index. Healthcare-adjusted model additionally adjusted for time-varying times spoken to a healthcare provider, multivitamins, calcium supplements, mammogram since prior visit. Blood pressure models additionally adjusted for blood pressure medication; cholesterol, triglyceride models additionally adjusted for statins.*

[2]*Among participants eligible to use blood pressure medication (n=624 at baseline).*

[3]*Hypertension status: "Never hypertensive" group has no history of antihypertensive use or measured hypertension; "pre-existing hypertension" group previously reported antihypertensive use or had measured hypertension; "new-onset hypertension" group reported antihypertensive use or had measured hypertension for the first time at the current visit.*

© 2024 Mitro SD et al. *JAMA Network Open.*

eTable 4. Longitudinal Discrete Survival Models Estimate the Risk of Newly Diagnosed Fibroids Associated With Each Cardiovascular Risk Factor in the Study of Women's Health Across the Nation (SWAN) Cohort. The "No fill" column represents regression models that are identical to the Healthcare-adjusted model but without carrying forward observations to fill random or structural missings (on average, 24% to 36% of values in main models were carried forward values, varying by cardiovascular exposure).

	Healthcare-adjusted[1]	No fill
**Characteristic**	HR (95%CI)	HR (95%CI)
**Blood pressure and medication**		
Systolic blood pressure (per 10-mm Hg)	1.01 (0.93, 1.10)	1.07 (0.98, 1.17)
Diastolic blood pressure (per 10-mm Hg)	0.98 (0.87, 1.12)	1.00 (0.87, 1.15)
Antihypertensive medication use (among eligible subset)[2]		
No antihypertensive use	*Reference*	*Reference*
Any antihypertensive use	0.63 (0.38, 1.05)	0.62 (0.34, 1.11)
ACE-inhibitor use	0.52 (0.27, 1.00)	0.50 (0.21, 1.16)
Hypertension status[3]		
Never hypertensive	*Reference*	*Reference*
Pre-existing hypertension	0.93 (0.73, 1.18)	0.93 (0.71, 1.22)
New-onset hypertension	1.45 (0.96, 2.20)	1.58 (1.03, 2.44)
Hypertension treatment		
No hypertension	*Reference*	*Reference*
Untreated hypertension	1.19 (0.91, 1.57)	1.18 (0.88, 1.60)
Treated hypertension	0.80 (0.56, 1.15)	0.75 (0.50, 1.15)
**Anthropometry**		
Body mass index (kg/m^2) (per 5-kg/m^2)	0.91 (0.77, 1.08)	0.85 (0.70, 1.03)
< 25.0	*Reference*	*Reference*
25.0 – 29.9	0.78 (0.59, 1.04)	0.78 (0.56, 1.08)
30.0 – 34.9	0.70 (0.48, 1.02)	0.68 (0.44, 1.04)
≥ 35	0.56 (0.33, 0.96)	0.45 (0.24, 0.86)
Waist circumference (per centimeter)	1.01 (0.99, 1.03)	1.00 (0.98, 1.02)
Waist-to-hip ratio (per 0.1-unit)	1.29 (1.02, 1.63)	1.29 (0.99, 1.69)
**Biomarkers**		
Total cholesterol (per 10-mg/dL)	0.98 (0.94, 1.02)	1.01 (0.96, 1.07)
LDL cholesterol (per 10-mg/dL)	0.99 (0.94, 1.04)	1.02 (0.95, 1.10)
HDL cholesterol (per 10-mg/dL)	1.03 (0.92, 1.16)	1.08 (0.91, 1.28)
Triglycerides (per 10-mg/dL)	0.99 (0.97, 1.01)	0.99 (0.96, 1.02)
C-reactive protein (per doubling)	1.01 (0.94, 1.09)	1.02 (0.92, 1.13)

[1]*Minimally-adjusted model adjusted for baseline value of the exposure, age, education, race/ethnicity, site; time-varying smoking status, menopausal status, parity, body mass index. Healthcare-adjusted model additionally adjusted for time-varying times spoken to a healthcare provider, multivitamins, calcium supplements, mammogram since prior visit. Blood pressure models additionally adjusted for blood pressure medication; cholesterol, triglyceride models additionally adjusted for statins.*

[2]*Among participants eligible to use blood pressure medication (n=624 at baseline).*

[3]*Hypertension status: "Never hypertensive" group has no history of antihypertensive use or measured hypertension; "pre-existing hypertension" group previously reported antihypertensive use or had measured hypertension; "new-onset hypertension" group reported antihypertensive use or had measured hypertension for the first time at the current visit.*

© 2024 Mitro SD et al. *JAMA Network Open.*

eTable 5. Longitudinal Discrete Survival Models Estimate the Risk of Newly Diagnosed Fibroids Associated With Each Cardiovascular Risk Factor in the Study of Women's Health Across the Nation (SWAN) Cohort. The "Includes post-menopause" column represents regression models that are identical to the Healthcare- adjusted model but without censoring at menopause (approximately 44% of observations were censored due to menopause in main models, ranging from 2% (visit 1) to 99% (visit 13)).

	Healthcare-adjusted[1]	Includes post-menopause
**Characteristic**	HR (95%CI)	HR (95%CI)
**Blood pressure and medication**		
Systolic blood pressure (per 10-mm Hg)	1.01 (0.93, 1.10)	1.01 (0.95, 1.08)
Diastolic blood pressure (per 10-mm Hg)	0.98 (0.87, 1.12)	0.98 (0.88, 1.09)
Antihypertensive medication use (among eligible subset)[2]		
No antihypertensive use	*Reference*	*Reference*
Any antihypertensive use	0.63 (0.38, 1.05)	0.71 (0.46, 1.10)
ACE-inhibitor use	0.52 (0.27, 1.00)	0.70 (0.42, 1.17)
Hypertension status[3]		
Never hypertensive	*Reference*	*Reference*
Pre-existing hypertension	0.93 (0.73, 1.18)	0.93 (0.75, 1.15)
New-onset hypertension	1.45 (0.96, 2.20)	1.49 (1.02, 2.16)
Hypertension treatment		
No hypertension	*Reference*	*Reference*
Untreated hypertension	1.19 (0.91, 1.57)	1.16 (0.91, 1.48)
Treated hypertension	0.80 (0.56, 1.15)	0.90 (0.67, 1.21)
**Anthropometry**		
Body mass index (kg/m^2) (per 5-kg/m^2)	0.91 (0.77, 1.08)	0.90 (0.78, 1.04)
< 25.0	*Reference*	*Reference*
25.0 – 29.9	0.78 (0.59, 1.04)	0.72 (0.56, 0.93)
30.0 – 34.9	0.70 (0.48, 1.02)	0.70 (0.51, 0.97)
≥ 35	0.56 (0.33, 0.96)	0.56 (0.36, 0.89)
Waist circumference (per centimeter)	1.01 (0.99, 1.03)	1.01 (0.99, 1.03)
Waist-to-hip ratio (per 0.1-unit)	1.29 (1.02, 1.63)	1.17 (0.96, 1.43)
**Biomarkers**		
Total cholesterol (per 10-mg/dL)	0.98 (0.94, 1.02)	0.98 (0.95, 1.02)
LDL cholesterol (per 10-mg/dL)	0.99 (0.94, 1.04)	0.98 (0.94, 1.03)
HDL cholesterol (per 10-mg/dL)	1.03 (0.92, 1.16)	1.01 (0.92, 1.12)
Triglycerides (per 10-mg/dL)	0.99 (0.97, 1.01)	1.00 (0.99, 1.01)
C-reactive protein (per doubling)	1.01 (0.94, 1.09)	1.02 (0.96, 1.09)

[1]*Minimally-adjusted model adjusted for baseline value of the exposure, age, education, race/ethnicity, site; time-varying smoking status, menopausal status, parity, body mass index. Healthcare-adjusted model additionally adjusted for time-varying times spoken to a healthcare provider, multivitamins, calcium supplements, mammogram since prior visit. Blood pressure models additionally adjusted for blood pressure medication; cholesterol, triglyceride models additionally adjusted for statins.*

[2]*Among participants eligible to use blood pressure medication (n=624 at baseline).*

[3]*Hypertension status: "Never hypertensive" group has no history of antihypertensive use or measured hypertension; "pre-existing hypertension" group previously reported antihypertensive use or had measured hypertension; "new-onset hypertension" group reported antihypertensive use or had measured hypertension for the first time at the current visit.*

© 2024 Mitro SD et al. *JAMA Network Open.*

eTable 6. Longitudinal Discrete Survival Models Estimate the Risk of Newly Diagnosed Fibroids Associated With Each Cardiovascular Risk Factor in the Study of Women's Health Across the Nation (SWAN) Cohort. The "Not adjusted for BL" column represents regression models that are identical to the Healthcare- adjusted model except that they are not adjusted for baseline values of the exposure.

	Healthcare-adjusted[1]	Not adjusted for BL
**Characteristic**	HR (95%CI)	HR (95%CI)
**Blood pressure and medication**		
Systolic blood pressure (per 10-mm Hg)	1.01 (0.93, 1.10)	0.99 (0.92, 1.06)
Diastolic blood pressure (per 10-mm Hg)	0.98 (0.87, 1.12)	0.95 (0.85, 1.06)
Antihypertensive medication use (among eligible subset)[2]		
No antihypertensive use	*Reference*	*Reference*
Any antihypertensive use	0.63 (0.38, 1.05)	0.69 (0.44, 1.07)
ACE-inhibitor use	0.52 (0.27, 1.00)	0.62 (0.36, 1.09)
Hypertension status[3]		
Never hypertensive	*Reference*	--
Pre-existing hypertension	0.93 (0.73, 1.18)	--
New-onset hypertension	1.45 (0.96, 2.20)	--
Hypertension treatment		
No hypertension	*Reference*	*Reference*
Untreated hypertension	1.19 (0.91, 1.57)	1.20 (0.93, 1.56)
Treated hypertension	0.80 (0.56, 1.15)	0.86 (0.66, 1.12)
**Anthropometry**		
Body mass index (kg/m^2) (per 5-kg/m^2)	0.91 (0.77, 1.08)	0.95 (0.88, 1.03)
< 25.0	*Reference*	*Reference*
25.0 – 29.9	0.78 (0.59, 1.04)	0.84 (0.65, 1.09)
30.0 – 34.9	0.70 (0.48, 1.02)	0.78 (0.57, 1.07)
≥ 35	0.56 (0.33, 0.96)	0.74 (0.54, 1.01)
Waist circumference (per centimeter)	1.01 (0.99, 1.03)	1.00 (0.99, 1.02)
Waist-to-hip ratio (per 0.1-unit)	1.29 (1.02, 1.63)	1.09 (0.93, 1.28)
**Biomarkers**		
Total cholesterol (per 10-mg/dL)	0.98 (0.94, 1.02)	0.99 (0.96, 1.02)
LDL cholesterol (per 10-mg/dL)	0.99 (0.94, 1.04)	1.00 (0.97, 1.03)
HDL cholesterol (per 10-mg/dL)	1.03 (0.92, 1.16)	1.01 (0.94, 1.09)
Triglycerides (per 10-mg/dL)	0.99 (0.97, 1.01)	0.99 (0.98, 1.01)
C-reactive protein (per doubling)	1.01 (0.94, 1.09)	0.98 (0.93, 1.04)

[1]*Minimally-adjusted model adjusted for baseline value of the exposure, age, education, race/ethnicity, site; time-varying smoking status, menopausal status, parity, body mass index. Healthcare-adjusted model additionally adjusted for time-varying times spoken to a healthcare provider, multivitamins, calcium supplements, mammogram since prior visit. Blood pressure models additionally adjusted for blood pressure medication; cholesterol, triglyceride models additionally adjusted for statins.*

[2]*Among participants eligible to use blood pressure medication (n=624 at baseline).*

[3]*Hypertension status: "Never hypertensive" group has no history of antihypertensive use or measured hypertension; "pre-existing hypertension" group previously reported antihypertensive use or had measured hypertension; "new-onset hypertension" group reported antihypertensive use or had measured hypertension for the first time at the current visit.*

© 2024 Mitro SD et al. *JAMA Network Open.*
