# Supplementary material for: Hypertension, Cardiovascular Risk Factors, and Uterine Fibroid Diagnosis in Midlife
Source: JAMA Netw Open. 2024 Apr 16;7(4):e246832. doi: 10.1001/jamanetworkopen.2024.6832 (PMC11022113; doi:10.1001/jamanetworkopen.2024.6832)
Supplement: Supplement 2. — Data Sharing Statement [file jamanetwopen-e246832-s002.pdf]

Data Sharing Statement

Mitro. Hypertension, Cardiovascular Risk Factors, and Uterine Fibroid Diagnosis in Midlife. *JAMA Netw Open*. Published April 16, 2024. doi:10.1001/jamanetworkopen.2024.6832

Data
Data available: Yes
Data types: Deidentified participant data, Data dictionary
How to access data: Deidentified SWAN data and complete data dictionary corresponding to this analysis are available for research following the approval of a research proposal by the Publications & Presentations Committee. Visits 0-10 are freely available to the public at the following website: https://www.swanstudy.org/swan-research/data-access/
When available: beginning date: 02-01-2014

Supporting Documents
Document types: None

Additional Information
Who can access the data: Deidentified SWAN data and complete data dictionary corresponding to this analysis are available for research following the approval of a research proposal by the Publications & Presentations Committee. Visits 0-10 are freely available to the public at the following website: https://www.swanstudy.org/swan-research/data-access/
Types of analyses: Deidentified SWAN data and complete data dictionary corresponding to this analysis are available for research following the approval of a research proposal by the Publications & Presentations Committee. Visits 0-10 are freely available to the public at the following website: https://www.swanstudy.org/swan-research/data-access/
Mechanisms of data availability: Deidentified SWAN data and complete data dictionary corresponding to this analysis are available for research following the approval of a research proposal by the Publications & Presentations Committee. Visits 0-10 are freely available to the public at the following website: https://www.swanstudy.org/swan-research/data-access/
